# Over Expression of Plk1 Does Not Induce Cell Division in Rat Cardiac Myocytes *In Vitro*

**DOI:** 10.1371/journal.pone.0006752

**Published:** 2009-08-25

**Authors:** Carmen H. Coxon, Katrina A. Bicknell, Fleur L. Moseley, Gavin Brooks

**Affiliations:** 1 School of Pharmacy, University of Reading, Reading, United Kingdom; 2 School of Biological Sciences, University of Reading, Reading, United Kingdom; 3 Department of Physiology, Anatomy and Genetics, University of Oxford, Oxford, United Kingdom; University of Minnesota, United States of America

## Abstract

**Background:**

Mammalian cardiac myocytes withdraw from the cell cycle during post-natal development, resulting in a non-proliferating, fully differentiated adult phenotype that is unable to repair damage to the myocardium, such as occurs following a myocardial infarction. We and others previously have shown that forced expression of certain cell cycle molecules in adult cardiac myocytes can promote cell cycle progression and division in these cells. The mitotic serine/threonine kinase, Polo-like kinase-1 (Plk1), is known to phosphorylate and activate a number of mitotic targets, including Cdc2/Cyclin B1, and to promote cell division.

**Principal Findings:**

The mammalian Plk family are all differentially regulated during the development of rat cardiac myocytes, with Plk1 showing the most dramatic decrease in both mRNA, protein and activity in the adult. We determined the potential of Plk1 to induce cell cycle progression and division in cultured rat cardiac myocytes. A persistent and progressive loss of Plk1 expression was observed during myocyte development that correlated with the withdrawal of adult rat cardiac myocytes from the cell cycle. Interestingly, when Plk1 was over-expressed in cardiac myocytes by adenovirus infection, it was not able to promote cell cycle progression, as determined by cell number and percent binucleation.

**Conclusions:**

We conclude that, in contrast to Cdc2/Cyclin B1 over-expression, the forced expression of Plk1 in adult cardiac myocytes is not sufficient to induce cell division and myocardial repair.

## Introduction

Mammalian cardiac myocytes lose the ability to divide shortly after birth, [1]–[4], differentiating into cell cycle arrested binucleated cells. Subsequent growth of the myocardium is achieved through an increase in myocyte cell size, known as hypertrophy. Following myocardial injury, cardiac myocytes are lost from the damaged area and replaced by scar tissue. Any loss in cardiac function is compensated by a physiological hypertrophic response and cardiac remodeling. Although this process initially helps to maintain cardiac output, the remodeling process continues and eventually leads to the development of pathological hypertrophy and heart failure [5]–[7].

The change in growth potential of cardiac myocytes during cardiac development and hypertrophy is reflected in the expressions and activities of certain cell cycle molecules.[8]–[15] Thus, positive regulators of the cell cycle machinery, such as the Cdk/cyclin complexes and the pro-proliferative E2F transcription factors (E2F1-3) are down regulated, whilst negative regulators, including retinoblastoma protein (pRb) and the cyclin-dependent kinase inhibitors (CDKIs), p21 and p27, are upregulated. As a consequence, the majority of adult mammalian cardiac myocytes are arrested in G_0_/G_1_, with the remaining cells being present at the G2/M transition[15].

Current strategies aimed at encouraging repair of the heart focus on the use of stem cells or gene therapy to induce cell division and differentiation at the site of damage to reduce the size of the infarct and improve patient outcome.[16], [17] In support of the gene therapy approach, work from our laboratory[12], [13], [18] and others (see below) have shown that manipulation of the cardiac myocyte cell cycle machinery could be a suitable therapeutic approach for promoting myocardial repair. Alterations in the expressions of certain cell cycle molecules can increase cardiac myocyte proliferation *in vitro*,[18]–[25] inhibit the induction of hypertrophy *in vitro* [12] or increase cardiac myocyte number[14], [24] and promote myocardial repair[25], [26] *in vivo*. These studies have highlighted a number of potential targets for the reinitiation of cardiac myocyte cell division, with the aim of increasing the number of cardiac myocytes and reducing infarct size to improve patient outcome.

The Polo-like kinases (Plks) are a family of serine/threonine kinases that exhibit sequence identity in both their N-terminal catalytic domain and their C-terminal region, which is termed the polo-box domain (PBD). This C-terminal region contains phosphopeptide binding motifs[27], [28], called ‘polo-boxes’, that are essential for Plk localization and regulation.[29]–[32] This family of kinases includes the mammalian Plks: Plk1, Plk2 (Snk), Plk3 (Fnk/Prk) and Plk4 (Sak-a/b); *Xenopus laevis* Plx1,[33] *Schizosaccharomyces pombe* plo1+ and *Saccharomyces cerevisiae* CDC5 [34]–[36].

Plk1 is important for mitotic progression (karyokinesis and cytokinesis); it is involved in the nuclear translocation of cyclin B1,[37] microtubule dynamics,[38]–[42] activation of Cdc25C,[42] inactivation of Wee1[43], [44] and Myt1,[45] separation of chromosomes at anaphase and destruction of mitotic proteins through activation of the anaphase promoting complex/cyclosome (APC/C).[46], [47] Recently, it has been demonstrated that Plk1 contributes to cytokinesis in human cells[48], [49] and that inhibition of Plk1 activity with the highly selective inhibitor, BI 2536,[48], [50], [51] results in cytokinesis failure and the generation of binucleated cells. Plk2–4 display varying roles in cell cycle progression: Plk2 plays a role in centriole duplication;[52] Plk3 is a non-cycling Plk[53] involved in G2 checkpoint signaling.[54] Plk4 differs from Plk1–3 as it contains a variation of the Plk kinase motif and has a single polo-box.[55] Plk4 has been shown to localize to centrioles and, like Plk2, is thought to be important for centriole duplication. Loss or over expression of Plk4 results in defects in the centriole cycle and aberrant spindle formation [56]–[59].

Previously, Plk1 mRNA and protein have been shown to decline during rat cardiac myocyte development.[60] We have extended these studies and characterized the expression of the whole family of mammalian Plks during rat cardiac myocyte development. We confirmed that the expression and activity of Plk1 in cardiac myocytes declines during development in accordance with observations previously reported.[60] Plk2, Plk3 and Plk4 also showed small, but significant, changes at the mRNA and protein levels during development. In addition, we have tested the hypothesis that over expression of Plk1 would reinitiate the cell cycle in G2/M-arrested cells and increase the number of cardiac myocytes. Adenovirus constructs were generated to express wild-type Plk1 or a constitutively active Plk1 with the phospho-mimicking T210D mutation. Despite being able to increase HeLa cell proliferation, we found that over expression of Plk1 was unable to induce cell cycle progression in isolated neonatal rat cardiac myocytes at 48 hours, or in adult rat cardiac myocytes at 24 hours. We found that this most likely due to the inability of Plk1 to increase the activity of Cdc2/cyclin B1. Based on these findings, we sought to further characterize the G2/M arrest originally observed in adult cardiac myocytes with the aim of determining a more precise description of the G2/M arrest. Using an immunohistochemical approach, we found no evidence of G2/M marker expression in the adult myocardium and conclude that this may be indicative of an earlier arrest.

## Materials and Methods

### Ethics Statement

Ventricular cardiac myocytes were isolated from wild type Wistar rats which were housed and maintained in a colony in accordance with the Local Ethical Review Panel of the University of Reading and U.K. Home Office guidelines. All experimental procedures using animals were performed in accordance with the U.K. Animals (Scientific Procedures) Act, 1986.

### Materials

Dual-labeled quantitative PCR probes and oligonucleotides were synthesized by Sigma-Genosys (Pampisford, U.K.). ABgene Absolute QPCR Rox Mix (ABgene; Epsom, UK) was used according to the manufacturer's instructions. Samples were normalized to either the ribosomal subunit, 18S, using the 18S QPCR kit (Applied Biosystems, Warrington, UK) or glyceraldehyde-3-phosphate dehydrogenase (GAPDH) QPCR kit (Applied Biosystems, Warrington, UK). Samples were amplified on an Applied Biosystems 5700 GeneAmp QPCR machine. The High-Fidelity PCR system was supplied by Roche Diagnostics (Lewes, U.K.). Anti-(cyclin B1) (GNS1) and anti-(CDC2 p34), antibodies were purchased from Santa Cruz Biotechnology (Santa Cruz, CA, U.S.A.). Anti-Plk1 monoclonal antibody cocktail was purchased from Zymed (Invitrogen, Paisley, U.K.). Anti-Plk2 and Plk4 antibodies were purchased from Bethyl Laboratories (Montgomery, TX, U.S.A.), and an anti-Fnk/Plk3 antibody was purchased from BD Biosciences. The anti-tropomyosin (sarcomeric) antibody (clone CH1) was obtained from Sigma (Poole, U.K.). Anti-Histone H3 Ser10 antibody was purchased from Millipore (Watford, U.K.) and anti-Histone H3 Ser28 and Ki67 antibodies were purchased from Abcam (Cambridge, U.K.) Horseradish-peroxidase-conjugated secondary antibodies were purchased from DakoCytomation (Ely, U.K.) and AlexaFluor® 568 and 488 anti-mouse IgG secondary antibodies were purchased from Molecular Probes (Eugene, OR, U.S.A.). Vectashield mounting medium containing DAPI (4′,6-diamidino-2-phenylindole) was purchased from Vector Laboratories (Orton Southgate, Peterborough, U.K.). Expression vector pIRES2-EGFP (enhanced green fluorescent protein) was obtained from BD Clontech UK (Basingstoke, U.K.). Quantity One® software package was from Bio-Rad (Hemel Hempstead, U.K.). BD Matrigel™ Matrix (growth factor reduced) was supplied by Fahrenheit Laboratories (Milton Keynes, U.K.). All other chemicals and biochemicals were supplied by Sigma.

### HeLa Cell Proliferation Assay

HeLa cells (5×105) were plated into 6 well dishes and maintained in DMEM supplemented with 10% FCS for 24 hours prior to infection. Cells were infected at an MOI of 50 and cell number was assessed by Coulter Counter at 48 hours post infection. Cells were washed, trypsinised and resuspended in Isoton II solution (Beckton Dickinson, Oxford, U.K.) prior to counting.

### Cardiomyocyte isolation

Fetal [gestational day 18 (E18)] and neonatal [2- (P2), 3- (P3), 5- (P5), 7- (P7) and 10- (P10) days old] ventricular cardiomyocytes were isolated from 30–70 excised ventricles of Wistar rats as described previously [12], [18]. Cells were maintained in either serum-free (0%) myocyte media (80% DMEM, 20% M199) or myocyte media supplemented with 5% fetal calf serum (FCS). Cardiomyocyte-enriched cultures were at least 95% pure, as determined by anti-tropomyosin staining. Adult ventricular cardiomyocytes were isolated from the excised hearts of male Wistar rats (175–200 g) by Langendorff perfusion [10], [13] and maintained in modified M199 media (M199 supplemented with 2 mM creatine, 2 mM carnitine and 5 mM taurine).

### Quantitative PCR

Primers and probes were designed using Primer Express™ Software (Applied Biosystems, Warrington, UK) as follows: Plk1 Rat Plk1 QPCR-Fwd 1121:5′- CTCCCGAACCACTGGTTCC-3′, Rat Plk1 QPCR-Rev 1189: 5′- CTTAATAAAGGTGTGGAGAACCCC-3′, Rat Plk1 Probe: 5′-TAMRATGCCTGACCGTCCCCGGAGAFAM- 3′; Plk2 QPCR-Fwd: 5′-GTCCTTTCAGTGGGTCACCAA-3′ Rat Plk2 QPCR-Rev: 5′- GTGTGGTCTGAGAGCTGGTATCC-3′, Rat Plk2 Probe: 5′-TAMRATGGGTCGACTACTCCAACAAATACGGCTT-FAM-3′; Plk3 QPCR-Fwd: 5′-GCCTACGCGGTCAAAGTCA-3′, Rat Plk3 QPCR-Rev: 5′- TTATGATCTTCTCGCGCTGATG-3′, Rat Plk3 Probe: 5′-TAMRACCGCAGAGTCGCGTCGCCAFAM- 3′; QPCR Rat Plk4-Fwd: 5′- AAGCAGAATGAGCGAGAATAG-3′, QPCR Rat Plk4-Rev: 5′- CCTTCCACTGTCACAGCAGAAG- 3′, Rat Plk4: 5′-TAMRACGGCTTTCCCAACACAGACACCAGTACTC-FAM-3′.

### Adenovirus-infection studies

Recombinant adenoviruses expressing wild-type (WT) or constitutively active Plk1 (T210D) or EGFP alone were generated using the AdEasy System (QBiogene, Carlsbad, USA). For adult cardiomyocyte infection studies, isolated adult myocytes were plated in serum-free modified M199 medium (M199 containing 2 mM creatine, 5 mM taurine and 2 mM carnitine) for 1 hr on Matrigel™-coated six-well dishes. Adherent adult cardiomyocytes were washed and then infected at a MOI (multiplicity of infection) of 50 plaque forming units of each virus per cell in modified M199 medium. Infected cultures were maintained in modified M199 medium for 24 h before analysis. Neonatal cells were transfected 24 hours after isolation. Cells were washed with PBS and infected in either serum-free M199 media or M199 supplemented with 5% FCS. Cells were incubated for 48 hours prior to analysis.

### Western Blotting

Protein expression levels were determined by SDS-PAGE analysis as previously described [10], [13].

### In vitro kinase assays

Cdc2/cyclin B1 immunocomplexes were immunoprecipitated from 250 µg of protein extracted from infected cultures using anti-cyclin B1 antibody and kinase activity measured as described previously [18]. For Plk1 kinase assays, protein lysate was prepared from infected cell cultures in TBSN lysis buffer (50 mM HEPES pH 7.4, 150 mM NaCl, 1 mM EGTA, 25 mM β-glycerophosphate, 25 mM NaF, 0.5 mM Na_3_VO_4_, 10 mM p-nitro phenylphosphate (pNPP), 0.1% Nonidet P-40, 72 µg/ml AEBSF, 10 µg/ml leupeptin, 5 µg/ml aprotinin). Samples were precleared with 30 µl Protein-G Agarose beads at 0.1 g/ml (Sigma) at 4°C on a rotating wheel for 1 hour. Plk1 was immunoprecipitated with 1.5 µl monoclonal mouse anti-Plk1 antibody cocktail (Zymed) and pulled down with 30 µl Protein G-Agarose. Immunoprecipitated Plk1 was washed twice in TBSN buffer and twice in TDSN (50 mM Tris pH 7.5, 10 mM MgCl_2_, 5 mM dithiothreitol (DTT), 2 mM EGTA, 0.5 mM sodium vanadate, 20 mM p-nitrophenyl phosphate) and incubated with 4 µg casein, 10 µCi AT32P and 10 µM ATP in 25 µl TDSN buffer at 30°C for 30 minutes. The reaction was terminated by boiling the samples in 1X Laemmli buffer with 0.2 M dithiothreitol (DTT) for 5 minutes. Samples were loaded on a 10% polyacrylamide gel and transferred onto nitrocellulose membrane. Membranes were assessed with a phosphorimager cassette (Molecular Dynamics) for the desired time.

### Immunocytochemistry

Neonatal cardiomyocytes were cultured in 5% myocyte media on gelatin-coated glass coverslips for 24 h before infection with recombinant adenoviruses. Adult cardiomyocytes were plated on to Matrigel™-coated chamber slides for 1 hr before infection with recombinant adenoviruses. Cell monolayers were fixed in 1% (v/v) formaldehyde in PBS for 10 min at room temperature (21°C), permeabilised with 0.5% (v/v) Triton X-100 in PBS for 15 min and blocked in 10% (v/v) normal goat serum/2% (w/v) BSA in PBS for 30 min. Cardiac myocytes were identified by staining with cardiac myocyte markers: anti-tropomyosin (sarcomeric) monoclonal antibody (clone CH1) was detected with anti-mouse IgG AlexaFluor® 568 or anti-troponin I was detected with anti-rabbit IgG AlexaFluor® 488 conjugate. These markers were used to distinguish cardiomyocytes from non-myocytes. Images were collected using an Axioskop2 microscope in conjunction with AxioVision software (Zeiss).

### Statistical analysis

All results are presented as means±standard error of the mean (SEM). Data were analyzed by one-way ANOVA followed by the appropriate t-test, with P<0.05 considered to be statistically significant.

## Results

### Expression and activity of Plk1 declines during rat cardiac myocyte development

**Figure 1:**
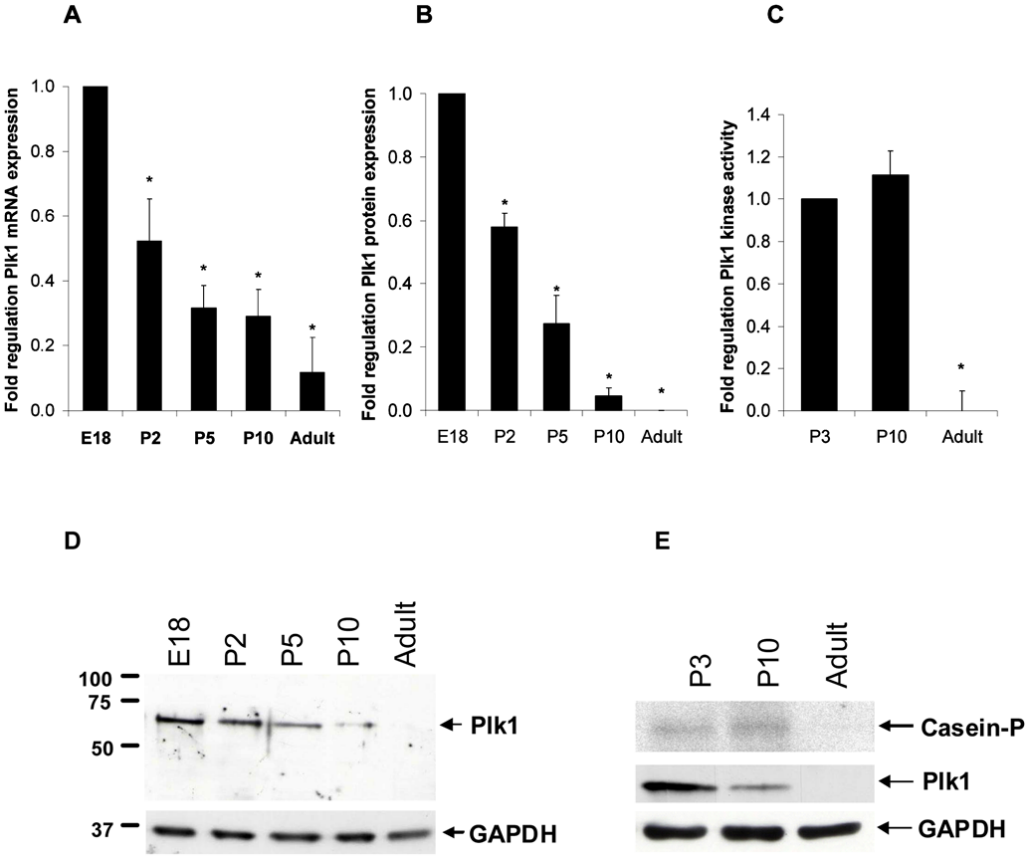
Plk1 expression and activity declines during rat cardiac myocyte development. Ventricular cardiac myocytes were isolated from Wistar rat hearts at various stages of development (embryonic day 18 [E18], 2, 5 and 10 days postnatal [P2, P5 and P10] and adult [250 g males]) and assessed for Plk1 mRNA (A, normalised to GAPDH) and protein (B, normalised to GAPDH) expression and activity (C). D) representative western blot of Plk1 expression. E) Representative kinase assay and corresponding protein expression (GAPDH was used as a loading control). Data is from three separate experiments, * p<0.05.

Based upon previous studies showing the downregulation of positive regulators of cell cycle molecules during cardiac myocyte development, we hypothesized that the expression of Plk1 would be downregulated as these cells progressed towards full differentiation. Quantitative PCR analysis revealed a progressive decline in Plk1 mRNA levels during development (Figure 1A) and this was also seen at the protein level (Figure 1B). No Plk1 protein was detected in adult cardiac myocytes. Interestingly, the activity of Plk1 does not change significantly at P10, compared to P3 levels, despite a decline in Plk1 expression (Figure 1B–E). These results suggest that there is a change in the regulation of Plk1 leading to increased activity at P10 compared to levels in P3 cardiac myocytes that is independent of protein synthesis.

### mRNA and protein levels of Plk2, Plk3 and Plk4 during rat cardiac myocyte development

**Figure 2:**
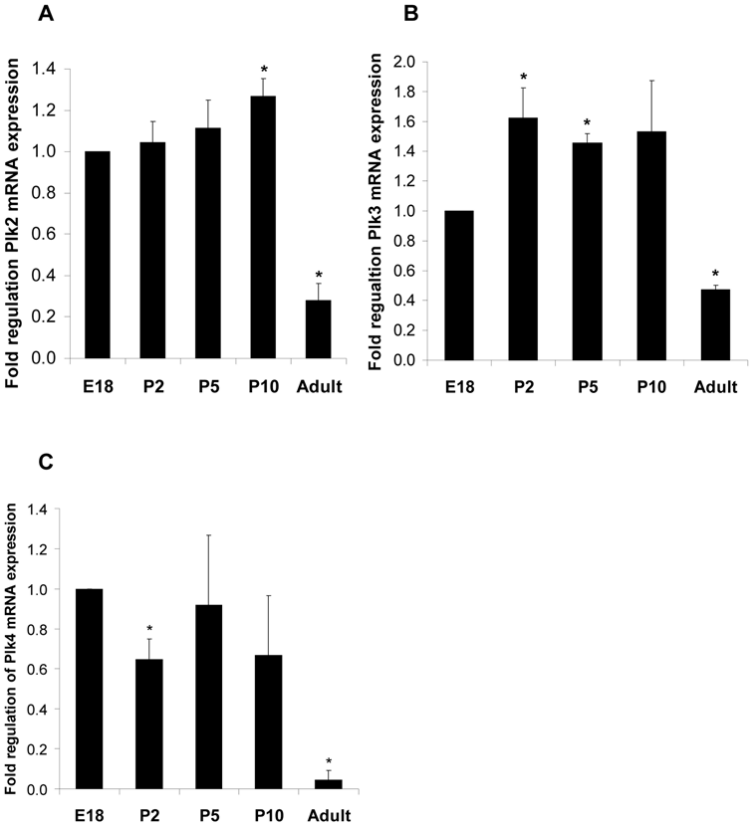
Regulation of Plk2, Plk3 and Plk4 mRNA expression during cardiac myocyte development. Ventricular cardiac myocytes were isolated from hearts at various stages of development (embryonic day 18 [E18], 2, 5 and 10 days postnatal [P2, P5 and P10] and adult [250 g males]) and assessed for Plk2, Plk3 and Plk4 mRNA (black bars, A, B and C, respectively). Samples were normalised to GAPDH. Each bar chart represents data from at least 3 separate experiments, * p<0.05.

**Figure 3:**
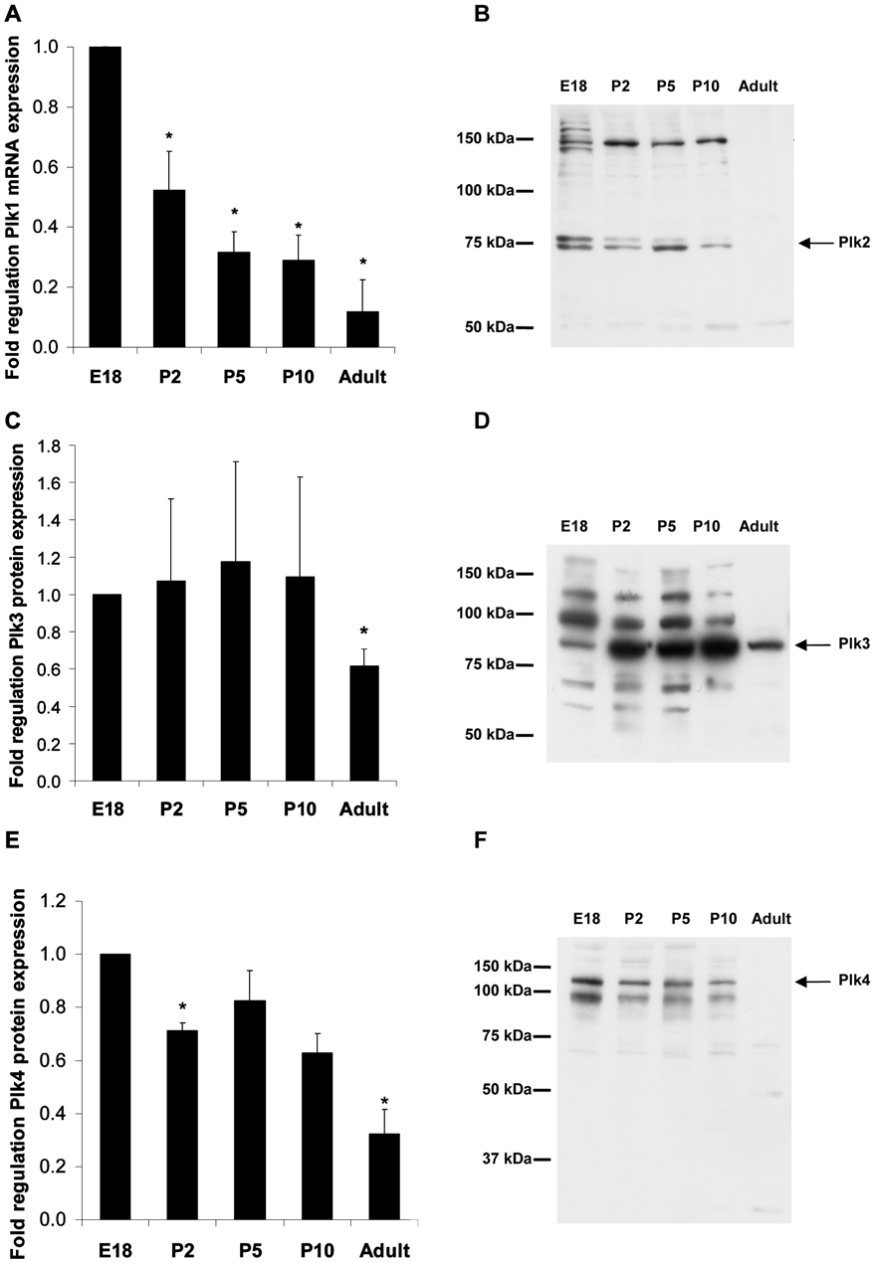
Expression of Plk2, Plk3 and Plk4 protein levels are regulated during cardiac myocyte development. Ventricular cardiac myocytes were isolated from hearts at various stages of development (embryonic day 18 [E18], 2, 5 and 10 days postnatal [P2, P5 and P10] and adult [250 g males]) and assessed for Plk2, Plk3 and Plk4 protein expression (A, C and E, respectively, black bars). Samples were normalised to GAPDH. Each bar chart represents data from at least 3 separate experiments, * p<0.05. Representative blots are shown for Plk2 (B), Plk3 ((D) and Plk4 (F).

Quantitative PCR revealed differential patterns of regulation for Plk2, Plk3 and Plk4 mRNA during the postnatal developmental period; all are significantly downregulated in the adult. Plk2 showed very little change from E18 levels throughout postnatal development (Figure 2A) whereas Plk3 showed a small, but significant, increase in mRNA levels during the early postnatal growth phase (P2 and P5) (Figure 2B). Plk4 showed a small, significant decrease at P2 and is downregulated by ∼90% in the adult compared to E18 levels (Figure 2C). At the protein level, Plk2, Plk3 and Plk4 are all expressed in the adult (Figures 3A to F), unlike Plk1, which is not be detected in adult cardiac myocytes (Figure 1B). Plk2 protein levels showed a considerable degree of variability between samples (n = 5); protein levels do not appear to change significantly during development. Plk3 protein expression also remains unchanged during early postnatal development, but does appear to be downregulated in the adult heart by ∼40% compared to embryonic day 18 (E18). Plk4 protein levels decrease as development proceeds, with a significant downregulation at P2 and in the adult (Figure 3E and F).

Based upon the data showed here, Plk1 is the most interesting candidate for re-initiation of cell cycle in adult cardiac myocytes. No protein expression or activity is detectable in the adult heart, whereas Plk2–4 are all expressed, albeit at lower levels, compared to E18.

### Adenoviral expression of active Plk1 in both HeLa cells and cardiac myocytes

**Figure 4:**
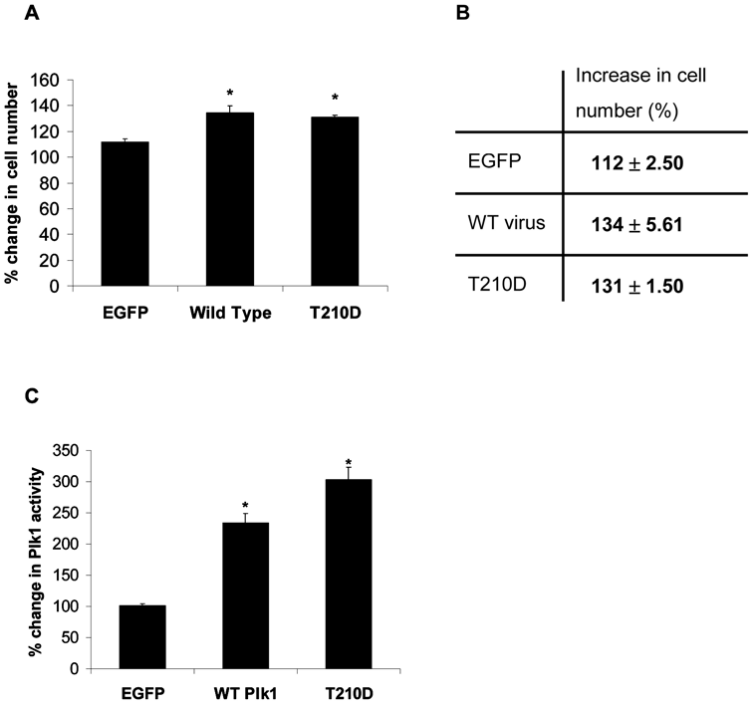
Effect of adenovirus-delivered Plk1 on HeLa cell number and Plk1 activity. Plk1 enhanced cell division in HeLa cells at 48 hours post infection (A and B). The activity of adenovirus-delivered Plk1 was assessed in P10 cardiac myocytes (C).

Petronczki *et al* (2007)[48] have shown that inhibition of Plk1 in HeLa cells released from a metaphase block results in binucleation. The majority of adult cardiac myocytes are binucleated and do not express Plk1. We hypothesized that reintroduction of Plk1 into isolated adult rat cardiac myocytes might drive the cells through a successful round of nuclear and/or cell division, thereby increasing cell number. Accordingly, we generated adenoviruses carrying Plk1 in conjunction with an internal ribosomal entry site (IRES) and EGFP marker. To confirm the ability of the Plk1 construct to induce cell cycle progression in proliferating cells, HeLa cells were infected with adenovirus containing either EGFP, wild-type or constitutively active (T210D) Plk1 at a multiplicity of infection (MOI) of 50 (50 virus particles per cell). Forty eight hours post infection, we found that over-expression of wild-type (WT) Plk1 increased the number of HeLa cells by 23±5.6% (n = 3, p = 0.02), compared to the EGFP control (Figure 4A and B). Constitutively active Plk1 (T210D) also increased HeLa cell numbers by 20±1.5% above the EGFP control. This data shows that adenovirus-delivered Plk1 was able to induce cell division in proliferative cells. We then assessed the activity of adenovirus-delivered Plk1 by *in vitro* kinase assay in non-proliferating P10 cardiac myocytes: Plk1 kinase activity was increased in both WT and T210D Plk1 expressing cells (Figure 4C).

### Over-expression of catalytically active Plk1 has no effect on cardiac myocyte binucleation

**Figure 5:**
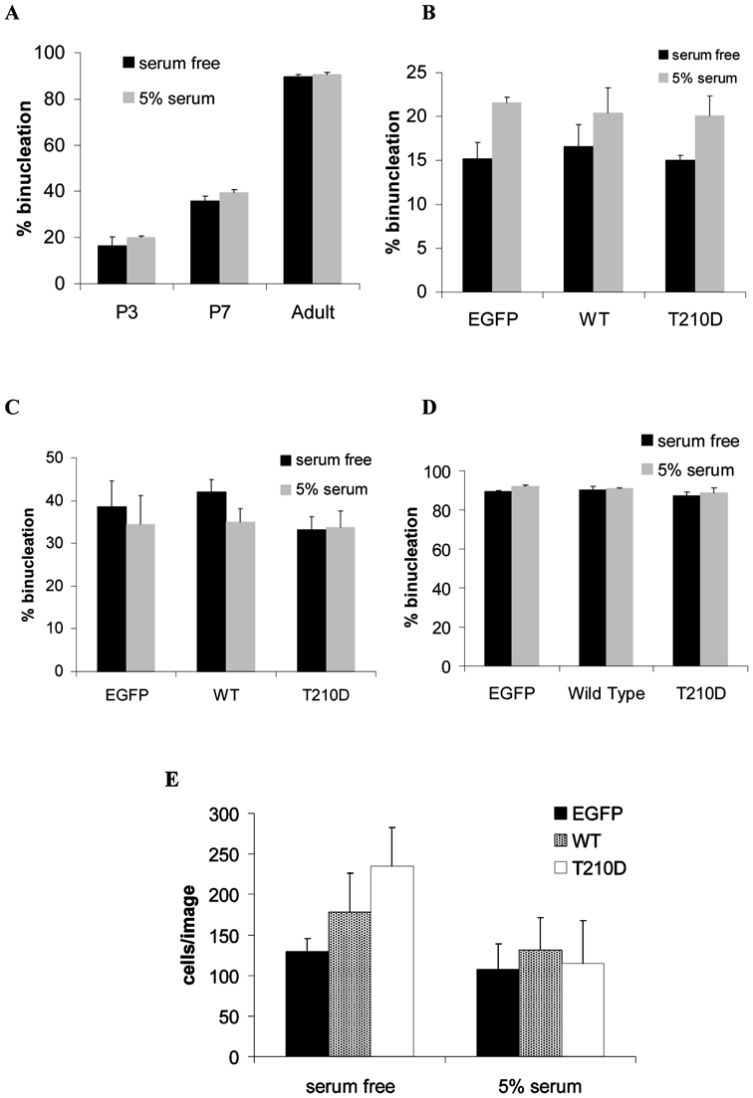
Over expression of Plk1 has no effect on adult cardiac myocyte binucleation. Ventricular cardiac myocytes were isolated and infected with EGFP, WT or T210D adenovirus and incubated for 48 hours (P3 and P7) or 24 hours (adult) in serum free media or media supplemented with 5% foetal calf serum (FCS). Cells were fixed and assessed for binucleation. A) Cardiac myocyte binucleation increases with age. Over expression of Plk1 had no effect on binucleation at P3 (B), P7 (C) or adult (D) stages of development. E) Plk1 over expression does not increase cardiac myocyte cell number, as determined by the number of troponin I positive cardiac myocytes per image (cells were counted for three separate images for EGFP, WT and T210D-infected cells from three separate experiments).

We next determined the effect of over expressing catalytically active WT and T210D Plk1 on cardiac myocyte cell binucleation. Ventricular cardiac myocytes were isolated from P3, P7 and adult Wistar rat hearts and subjected to adenovirus infection as above; P7 cardiac myocytes were chosen due to their improved viability following isolation compared to P10 cells (data not shown). The effect of EGFP, WT or constitutively active (T210D) Plk1 expression on cardiac myocyte binucleation was assessed by calculating the number of nuclei per cardiac myocyte. For uninfected postnatal cells, the number of binucleated cells increased from ∼20% at P3 to ∼40% at P7. Adult cardiac myocytes showed extensive (∼90%) binucleation (Figure 5A). Surprisingly, we found adenovirus-delivered Plk1 did not have a significant effect on the percentage of binucleated cardiac myocytes at any stage of development (Figures 5B, C and D). This was somewhat surprising, especially at P3 where there is still a capacity for cell division. To determine whether there had been an effect on cell number but not on binucleation, we performed cell counts at P3 and found that there was no significant increase in cardiac myocyte cell number when the cells were incubated in either serum free media (n = 3, WT p = 0.378; T210D p = 0.100) or media supplemented with 5% FCS (n = 3, WT p = 0.277, T210D p = 0.892) compared to EGFP control (Figure 5E). Cell counts were performed on permeabilised, fixed samples stained with cardiac specific markers so rule out increases in cell number by contaminating cardiac fibroblast cells.

### Over-expression of Plk1 does not increase the activity of Cdc2/cyclin B1 in P3 cardiac myocytes

**Figure 6:**
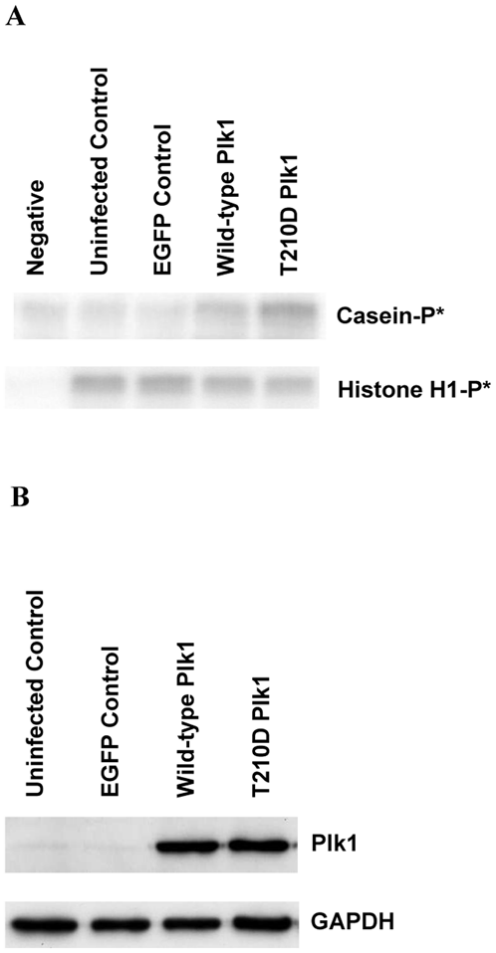
Exogenous Plk1 does not increase the activity of endogenous Cdc2/cyclin B1 in isolated P3 rat cardiac myocytes. Ventricular cardiac myocytes were isolated from Wistar rats 3 days after birth (P3). Adenovirus expressing EGFP, WT or T210D was used to infect cells for 48 hours. Cells were harvested and assessed for Plk1 and Cdc2/cyclin B1 kinase activity. Plk1 activity was assessed by its ability to phosphorylate casein (A, upper panel). Cdc2/cyclin B1 activity was assessed by phosphorylation of histone H3 (A, lower panel). B) Representative western blot of Plk1 over expression in P3 cardiac myocytes infected with EGFP, WT or T210D adenovirus. GAPDH was used to verify equal loading.

Whilst over-expression of Plk1 had no effect on binucleation in adult cardiac myocytes, it was surprising that Plk1 had no effect on P3 cardiac myocytes at all. P3 cardiac myocytes express the mitotic regulator Cdc2/cyclin B1[18] and retain a limited proliferative capacity. Plk1 promotes activation of Cdc2/cyclin B1[37], [42] and inhibits the activity of the Cdc2/cyclin B1 inhibitors, Wee1 and Myt1[43], [45], [61]. Therefore, we evaluated the effect of Plk1 over expression on Cdc2/cyclin B1 activity in P3 rat cardiac myocytes to determine whether this might explain the lack of cell cycle progression. Interestingly, the activity of Plk1 increased in cells infected with both wild-type and constitutively active Plk1 adenovirus, whereas the activity of Cdc2/cyclin B1 in these cells was unaffected (Figure 6). This data shows that the activation of Cdc2/cyclin B in differentiating rat cardiac myocytes cannot be achieved solely by the over expression of Plk1 and highlights the complexity of cell cycle regulation in cardiac myocytes. This work shows that careful consideration is required when choosing which molecules should be selected for these studies. It is clear that the cell cycle must be manipulated in precisely the right way in order to elicit cell cycle progression.

### Cardiac myocytes do not express G2/M markers

The current study aimed to determine whether Plk1 could be a potential therapeutic target for the initiation of cardiac repair in the mammalian heart. This hypothesis was based on previous studies that have shown that a significant population of adult cardiac myocytes is G2/M arrested [14], [15], [62], [63]], and that over expression of the G2/M regulator Cdc2/cyclin B1 is sufficient to increase cell number *in vitro* [18]. Although the data presented here shows that Plk1 is unable to do this, it does highlight that a more definitive understanding of the arrest in cardiac myocytes is necessary in order to successfully identify potential targets for a gene therapy approach. In order to further characterise the arrest in cardiac myocytes, and a therefore provide a smaller number of potential targets, we decided to more precisely define the point within the G2/M arrest that these cells are arrested in. Four micrometre sections were taken from E18, P3, P10 and adult Wistar rat hearts (Figure 6) and assessed for the expression of Ki67 and phosphorylation of histone H3 at serine 10 (Ser10) and at serine 28 (Ser28).

The proliferation marker, Ki67, is present in all phases of the cell cycle, increasing from G1 to maximal intensity at the metaphase-anaphase transition, but is not found in telophase nor in quiescent, G0, cells[64]. Histone H3 becomes phosphorylated on Ser10 during late G2 and is important for chromosome condensation[65]–[67]. Ser28 is also phosphorylated during mitosis, and like Ser10 phosphorylation, contributes to chromosome condensation [68]. However, unlike Ser10, Ser28 is not phosphorylated until the onset of mitosis[68] and therefore serves as a more faithful mitotic marker.

**Figure 7:**
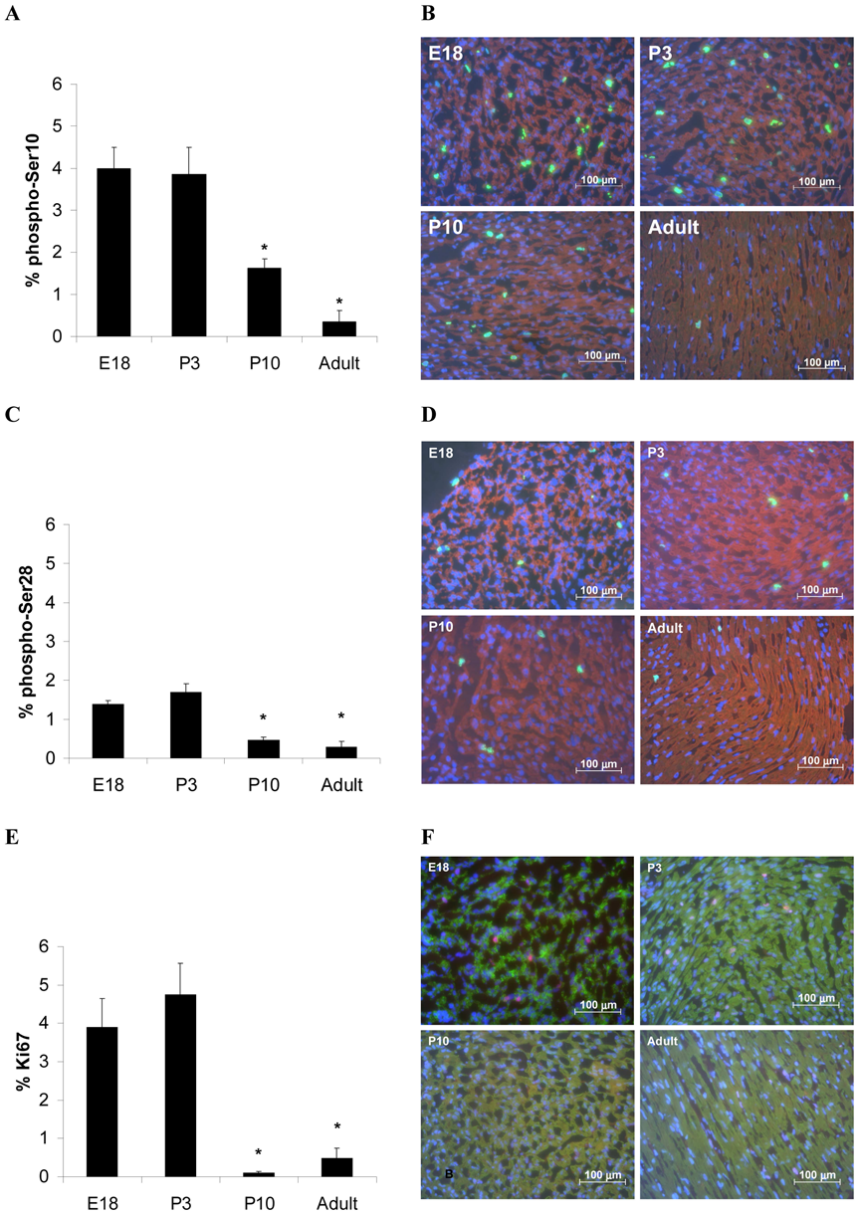
Loss of cell cycle markers from the developing rat heart correlates with the decline in proliferative capacity of the cardiac myocyte. Hearts were excised from embryonic (E18), neonatal (P3 and P10), and adult Wistar rats, frozen in embedding media and sections were cut at 4 µm. Left hand panel (A, C and E) show bar chart summaries (n = 3, SEM, * p<0.05) while the right hand panel (B, D and F) show representative sections for each cell cycle marker at each age group. (B) Cardiac myocytes were identified by the cardiac myocyte-specific marker, tropomyosin (red), histone H3 phosphorylated at Ser10 (green), and nuclei were stained with DAPI (blue). (D) Cardiac myocytes were identified by the cardiac myocyte-specific marker, tropomyosin (red), histone H3 phosphorylated at Ser28 (green), and nuclei were stained with DAPI (blue). (F) Cardiac myocytes were stained with the cardiac myocyte specific marker, troponin I, Ki67 staining is red and DNA was stained with DAPI (blue).

The expression of Ki67 and the phosphorylation of histone H3 at Ser10 and Ser28 declined with age (Figure 7). Very few (Ki67, 0.49±0.25%; Ser10, 0.35±0.27%; Ser28, 0.29±0.15%; n = 3, mean±SEM) troponin-I-positive adult cardiac myocytes demonstrated any staining or mitotic figures. Previous studies have investigated the isolated nuclei of adult cardiac myocytes using FACS analysis using dual staining for bromodeoxyuridine (BrdU) and propidium iodide (PI) [14], [15]. This type of analysis determines cell cycle stage by assessing the DNA content of each event (cell or nucleus) as it passes through the laser. Any event displaying a double DNA content, such as a post-S phase, mitotic or binucleated cell, will be classified as a G2/M cell. The lack of any Ser10 or Ser28 staining or mitotic structures suggests that the G2/M population originally observed in adult rat cardiac myocytes most likely represents a population of late S-phase/early G2-arrested cells, rather cells in late G2/M. This might explain why Plk1 is unable to induce cell cycle progression while Cdc2/cyclin B1 can; Cdc2/cyclin B1 reaches its peak activity before the G2/M transition while Plk1 peaks at metaphase[69].

## Discussion

In this study, we have determined the expression of the mammalian Plk family of serine/threonine protein kinases in the developing rat cardiac myocyte in an effort to better understand why adult cardiac myocytes are unable to proliferate and repair the damaged myocardium, and to potentially identify novel therapeutic targets that could be manipulated to encourage myocardial repair.

Developing cardiac myocytes undergo binucleation during neonatal growth,[70]–[73] whilst adult cells undergo a G1/S transition during the process of hypertrophy.[2], [74]. Both Plk2 and Plk4 are required for S-phase progression and the associated centriole duplication process [52], [56]–[59] and this most likely explains the presence of these two proteins in the adult cardiac myocyte.

Plk3 was originally identified as an immediate-early response gene that was upregulated in NIH3T3 cells in response to FGF.[75] It has been reported that Plk3 has a role in G2 checkpoint signaling [76], [77] and is activated following treatment of cells with 100 µM doxorubicin or H_2_O_2_.[54], [78] We show here that at the protein level, the expression of Plk3 does not change during rat cardiac myocyte development, with levels persisting to the adult stage. Adult cardiac myocytes are thought to be blocked in G2/M and it is possible that Plk3 could contribute to the arrested state of these cells.

The decline in Plk1 mRNA and protein levels and activity observed during cardiac myocyte development is consistent with previous studies from our laboratory, and others, examining the expression of other such positive regulators of the cell cycle[8], [11], [12], [18], [21], [23]–[25] and extends the work by Georgescu *et al* [60]. To investigate the role of Plk1 in nuclear and cellular division in rat cardiac myocytes, we over expressed wild type and constitutively active Plk1 in adult myocytes for 48 hours in the absence or presence of serum to determine whether Plk1 activity could induce cell division. Neither the wild type nor the constitutively active mutant induced any changes in cell cycle progression, in terms of the percentage of binucleated cells, or cell number. Surprisingly, we also found that catalytically active Plk1 was unable to increase the activity of endogenous Cdc2/cyclin B1 in P3 cardiac myocytes, suggesting that Plk1 is unable to complete with the other factors that regulate activity of the Cdc2/cyclin B1 complex. As well as dephosphorylation of Cdc2 at Thr14 and Tyr15 by Cdc25C, phosphorylation of Thr161 in the catalytic T-loop by Cdk activating kinase (CAK) is essential for Cdc2/cyclin B1 activity.[79] It is likely that some of these other factors limit the activation of endogenous Cdc2/cyclin B1; for example, CAK phosphorylates Cdc2 at Thr161 [80] and this modification is required for full activation of the Cdc2/cyclin B1 complex. If Plk1 can only enhance the activity of Cdc2/cyclin B1 that already is phosphorylated at Thr161, the amount of active Plk1 present would be irrelevant if CAK is the rate-limiting factor in Cdc2/cyclin B1 activation. In addition to this, whilst Plk1 has been reported to inhibit the Cdk inhibitor, Wee1, it has been shown that an initial Cdk-mediated phosphorylation of Wee1 at Ser121 is necessary to produce a Plk1 docking site, and that this phosphorylation event is required for increased Plk1 binding[43]. If the Cdk activity in P3 cardiac myocytes is not sufficient to prime Wee1 for Plk1 binding, over expression of Plk1 alone will not be sufficient for Wee1 inhibition and, therefore, an increase in Cdc2/cyclin B1 activity.

Cdc2/cyclin B1 is involved in some of the early stages of mitosis, such as the breakdown of the nuclear envelope and chromosome condensation[81]. Because ectopic Cdc2/cyclin B1 can elicit an increase in the number of adult cardiac myocytes, whilst Plk1 can not, it is possible that in the heart these cells are in an early/mid-G2, rather than in late-G2/M arrest. As such, the introduction of Plk1 into adult myocytes could be too far downstream of this arrest, and therefore unable to bypass the other events that are necessary to occur before mitosis can take place. Based upon the observations reported here, we sought to characterize the G2/M arrest in more detail. Previous reports have suggested that a G2/M arrested population of cardiac myocytes exists in the rat adult heart.[14], [15] To examine this further, we prepared heart sections and assessed them for any evidence of mitotic arrest by the phosphorylation of histone H3 on serine 10 or serine 28. Cells staining positive for cardiac myocyte markers and phosphorylation of either serine residue declined with age, with virtually no adult cardiac myocytes staining positive. The expression of the cell cycle marker, Ki67, was also assessed during rat heart development and was shown to be absent from the adult heart. This data suggests that adult cardiac myocytes are not in a state of ‘G2/M’ arrest as previously reported[15], but that the arrest could be earlier, such as late S/G2. This raises questions about the involvement of mechanisms of endoreduplication, rather than mitosis, being responsible for cell cycle arrest in the adult cardiac myocyte. Cardiac myocytes are known to increase in terms of ploidy following myocardial infarction[82]. It is possible that the increase in ploidy seen in the myocardium surrounding an infarct[82] is an attempt at cell division that is inhibited by the absence of Cdk or Plk1 activity at G2, leading to rounds of DNA synthesis in the absence of cell division. This may partially explain the presence of the previously reported population of G2/M nuclei;[15] these nuclei may in fact be the product of replication origin re-licensing and endoreduplication rather than an aborted mitosis.

It is vital that this arrest is characterised in more detail; identifying the precise site of the arrest is important if we are to successfully find new targets for re-initiating cardiac myocyte division post-infarct or during heart failure.
